# Genetic algorithm-based daily power output forecasting for energy storage power stations

**DOI:** 10.1371/journal.pone.0342331

**Published:** 2026-04-24

**Authors:** Lingzhi Xi, Jianxu Zhong, Shaofeng Yu, Chongyang Liao, Jinpeng You

**Affiliations:** Information and Communication Branch, China Southern Power Grid Energy Storage Co. Ltd, Guangzhou, Guangdong, China; University of Lagos Faculty of Engineering, NIGERIA

## Abstract

Accurate day-ahead power generation forecasting is crucial for improving the operational efficiency of energy storage power stations and enhancing the reliability of power grid dispatch. To address the challenges of prediction inaccuracy stemming from the complex nature of energy storage power stations, the difficulties in quantifying charge-discharge energy losses, and the obstacles in extracting implicit features from historical power data, this paper proposes a hybrid forecasting method that integrates chaos theory, signal decomposition, and deep learning, optimized using an adaptive genetic algorithm. First, a refined loss model is established for the battery packs, power conversion systems (PCS), transformers, and station auxiliary power consumption, to quantify energy losses during operation. Second, chaotic phase-space reconstruction is applied to the historical power generation data to reveal its inherent dynamic characteristics. Subsequently, the reconstructed sequences are processed using the Ensemble Empirical Mode Decomposition (EEMD) method to obtain a series of Intrinsic Mode Function (IMF) components. Based on these components, a Peak-based Frequency Band Division (PFBD) method is employed to aggregate the IMFs into high-frequency and low-frequency feature components, thereby effectively extracting implicit information from the original power sequences. Subsequently, a hybrid forecasting model integrating a Convolutional Neural Network (CNN), a Long Short-Term Memory Network (LSTM), and a Multi-Layer Perceptron (MLP) – denoted as CNN-LSTM-MLP – is constructed. This model takes as its input a combination of the extracted implicit features and the outputs from the loss model. The CNN captures local spatial patterns, the LSTM learns long-term temporal dependencies, and the MLP performs feature integration and nonlinear mapping. Finally, an Adaptive Genetic Algorithm (AGA) is used to automatically optimize the hyperparameters of the hybrid model, thereby improving forecasting performance. Experiments using actual operational data from a 10 MW/20 MWh electrochemical energy storage power station demonstrate that the proposed method achieves excellent performance in day-ahead 24-hour forecasting. The Mean Squared Error (MSE), Mean Absolute Error (MAE), and Coefficient of Determination (R²) reach 6.41 MW², 7.68 MW, and 0.898, respectively. Both forecasting accuracy and goodness of fit significantly outperform those of the compared single or hybrid models. This study provides an effective solution that integrates data-driven approaches with physical models for accurate power forecasting in energy storage power stations.

## 1. Introduction

Under the promotion of global energy transformation and the “dual carbon” goal, the transformation of energy structure is accelerating, and the importance of new energy such as solar energy and wind energy is highlighted [1]. However, renewable energy generation faces challenges of intermittency and volatility [2,3], such as solar dependence on sunlight, wind power generation being affected by wind speed [4], and difficulty in predicting and controlling power output [5], posing a threat to the safety of the power system. Energy storage facilities are the key solution [6], which can smooth power generation fluctuations, improve output stability, and flexibly allocate power across time periods and regions, enhancing system resilience and reliability [7]. Accurately predicting the power generation of energy storage stations is beneficial for advance planning and scheduling of power generation, optimizing resource allocation, improving power supply stability and quality, reducing interruptions, and ensuring power safety [8].

To accurately predict the power generation of power plants, researchers have tried various methods. Shin et al. used STL to process multidimensional data and predicted photovoltaic power generation using XGBoost. However, due to poor coupling of multidimensional data, XGBoost ignoring local spatiotemporal features, and weak adaptability to extreme weather data, there are problems such as information loss [9]. Tabbasum et al. obtained multidimensional data from IoT sensors and used DNN combined with SMA for edge node prediction. Although SMA performs well in global search, DNN carries gradient risk and requires a large amount of high-quality data. SMA converges slowly in the later stage and is prone to local optima [10]. Souhe et al. constructed a GRU-SVM model, where GRU captures the long and short variation features of photovoltaic data and SVM generates predictions, but insufficient spatial features are extracted, which affects the prediction [11]. Khan et al. developed a DSPM model that can simultaneously extract spatiotemporal features, dynamically allocate weights, capture causal relationships, and make individual predictions. However, in practical applications, performance may be affected by “task interference” and the risk of overfitting may increase [12]. Abedinia et al. used a one-dimensional convolutional network combined with LSTM to adaptively map quantile functions for predicting wind power, but relied on a single data type, resulting in poor adaptability and large errors [13].

In addition, for the specific subject of energy storage power stations, prediction research also needs to pay particular attention to the precise modeling of internal system losses. Most existing studies treat energy storage power stations as “black boxes” or simplified models, overlooking the non-linear losses of equipment such as battery packs, Power Conversion Systems (PCS), and transformers during the charging and discharging processes, which vary with time, load, and temperature. This directly impacts the prediction accuracy targeting net power [14]. Meanwhile, at the level of feature engineering, most methods directly use the original power sequences or simply incorporate external features, failing to effectively mine the inherent dynamic laws, potentially with chaotic characteristics, from the single-variable power historical data generated by the operation of energy storage power stations themselves, which contains rich information about system states [15]. In terms of model optimization, deep learning models have numerous hyperparameters, making manual tuning difficult and prone to getting stuck in local optima. Traditional methods such as grid search entail high computational costs and lack efficient optimization mechanisms for high-dimensional non-convex problems [16].

Genetic algorithms, as optimization algorithms that simulate natural selection and genetic mechanisms, possess advantages such as strong global search capabilities, robustness, and suitability for complex optimization problems. They can conduct extensive searches in the solution space, avoiding getting trapped in local optima and thus finding solutions closer to the global optimum. Applying genetic algorithms to day-ahead power generation forecasting for energy storage power stations holds the potential to fully leverage their optimization search advantages. However, how to deeply integrate them with a hybrid prediction framework capable of simultaneously modeling spatiotemporal features, internal losses, and chaotic dynamics to achieve end-to-end performance improvements from feature extraction, model construction to parameter optimization remains an unexplored research gap. In view of this, this study proposes a hybrid prediction framework optimized by an adaptive genetic algorithm, aiming to systematically address the aforementioned challenges. The main contributions of this paper include:

(1) Constructing a refined loss model for electrochemical energy storage power stations and incorporating it as a physical constraint into the data-driven prediction framework;
(2) Proposing a feature extraction method that combines chaotic phase-space reconstruction, Ensemble Empirical Mode Decomposition (EEMD), and Peak-based Frequency Band Division (PFBD) to adaptively extract implicit features representing multi-scale system dynamics from single-variable power sequences;
(3) Designing a CNN-LSTM-MLP hybrid neural network to collaboratively capture the spatiotemporal dependencies and non-linear relationships of input features;
(4) Employing an adaptive genetic algorithm to automatically perform global optimization of key hyperparameters of the hybrid model to enhance prediction accuracy and generalization ability.

Through practical case validation, the proposed method in this paper provides a new approach for high-precision day-ahead power forecasting in energy storage power stations.

## 2. Day-ahead power output forecasting for energy storage power plants

### 2.1. Analysis of energy consumption characteristics in energy storage power stations

**Fig 1:**
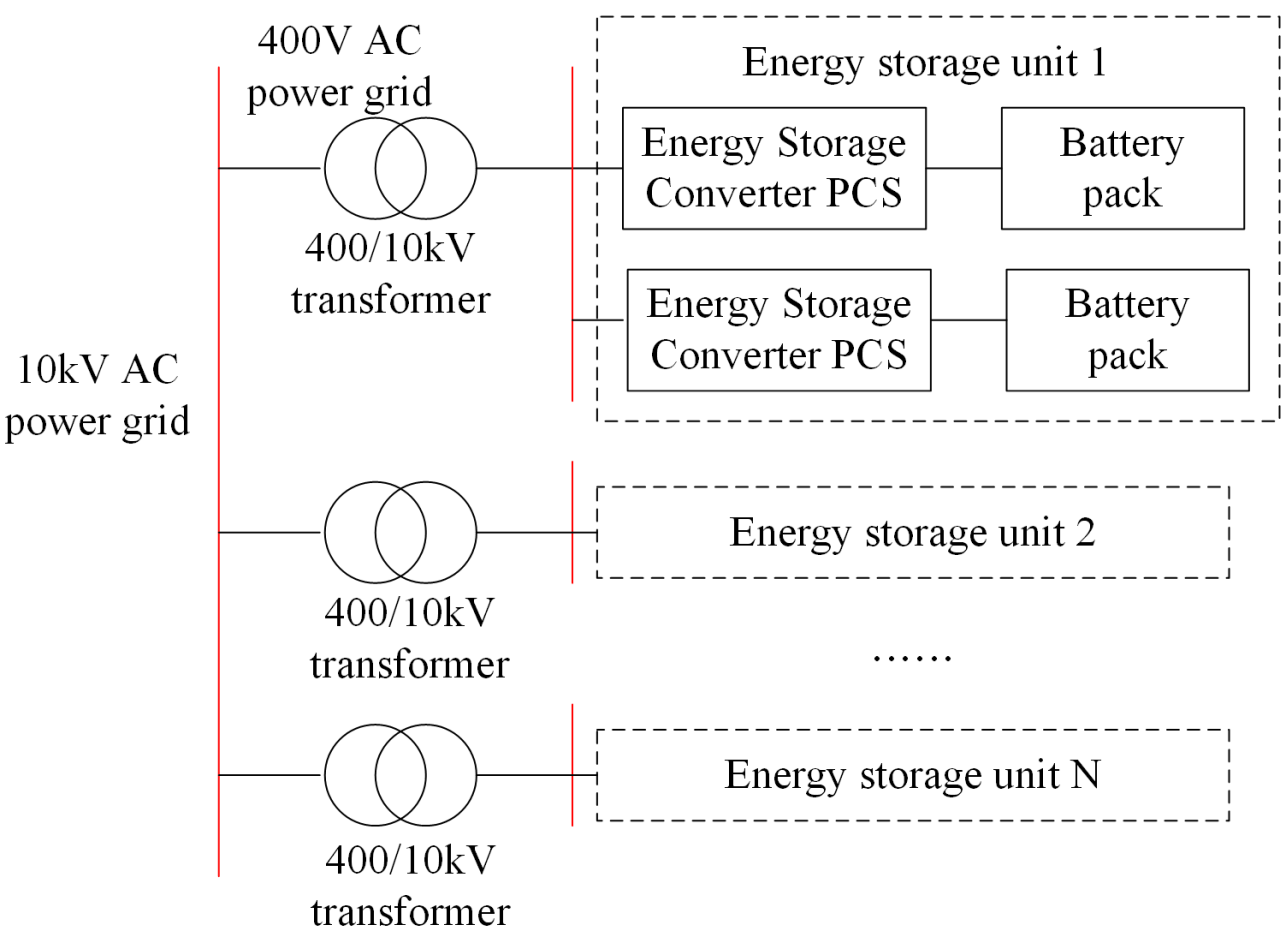
Topology diagram of energy storage power station.

Energy consumption analysis of energy storage power stations serves as the foundation for accurately predicting their net power generation. The losses mainly arise from the physical processes of electrical energy conversion and transmission, specifically occurring in the battery pack, power conversion system (PCS), step-up transformer, as well as auxiliary equipment (station power consumption) that maintains the operation of the power station. Fig 1 illustrates the topological structure of a typical electrochemical energy storage power station. Quantitative modeling of these losses aids in more accurately deducting losses from the “gross power” during prediction, thereby obtaining a “net power” prediction value that is closer to the measurement point at the grid interface.

#### 2.1.1. Energy storage unit loss model.

The energy storage unit is the core unit for charging and discharging, and its losses primarily stem from the internal heat generation of the battery pack and the conversion losses of the PCS.

(1) Battery Pack Loss Model

When a battery is being charged or discharged, the current flowing through its internal resistance generates Joule heat, which constitutes the main part of battery losses. This loss is closely related to the battery’s series-parallel configuration, state of charge (SOC), state of health (SOH), and the magnitude of the charging/discharging current. Within the engineering applicable range (e.g., when SOC is between 20% and 90%), the model of a single battery cell can be simplified as a series connection of an ohmic internal resistance $R_{o}$ and a polarization internal resistance $R_{p}$. For a battery pack composed of a single cells connected in series and then o such series groups connected in parallel, its total power loss $P_{LBatt}$ can be calculated using the following formula:


$$\left\{ \begin{array}{c} P_{LBatt}=oP_{LBatt0}\\ P_{LBatt0}=\left(R_{o}a+R_{p}\alpha_{con}\alpha_{SOC}\alpha_{T}\right)\overset{2}{\underset{DCB}{I}}+\alpha_{LBatt}a\\ \eta_{Batt}=1-P_{LBatt}/\left|P_{BATT}\right| \end{array}\right.$$ (1)

Among them, $P_{LBatt}$ is the power loss of the battery pack, $P_{LBatt0}$ is the basic power loss (without correction factor), o is the number of parallel battery strings, a is the number of batteries per string, $R_{o}$ and $R_{p}$ are the ohms and polarization resistance of individual batteries, $I_{DCB}$ is the current in the string, $\alpha_{LBatt}$ is the zero current loss, $\alpha_{con}$ is the configuration correction coefficient, $\alpha_{SOC}$ is the SOC correction coefficient, $\alpha_{T}$ is the working state correction coefficient, $\eta_{Batt}$ is the charging and discharging efficiency, and $P_{BATT}$ is the output power. The above three correction coefficients are obtained through fitting experimental data and are used to correct the additional losses caused by connection impedance, nonlinear electrochemical characteristics, and differences in charge-discharge efficiency during actual operation.

(2) Power Conversion System (PCS) Loss Model

The losses of the PCS mainly include switching device losses and passive filter losses. Its efficiency is expressed as a function of the load rate and can be obtained from the manufacturer’s datasheet or through experimental measurements to acquire typical curves. Therefore, the efficiency of the entire energy storage subsystem (battery pack + PCS) can be approximately regarded as the product of the battery pack efficiency and the PCS efficiency:


$$\eta_{sub}=\eta_{Batt}\eta_{PCS}$$ (2)

Among them, $\eta_{sub}$ is the energy storage subsystem efficiency; $\eta_{PCS}$ is the energy storage converter efficiency.

#### 2.1.2. Transformer and station power loss model.

(1) Transformer Loss Model

The losses in a transformer mainly consist of copper losses, which vary with the load, and iron losses, which are related to the core material and frequency.

Copper losses are caused by the resistances of the primary and secondary windings. The calculation formula is as follows:


$$P_{Closs}=R_{cp}\overset{2}{\underset{p}{I}}+R_{s}\overset{2}{\underset{s}{I}}$$ (3)

Among them, $R_{cp}$ and $R_{s}$ are the primary and secondary equivalent AC resistances, $I_{p}$ and $I_{s}$ are the primary and secondary side currents.

Iron losses are caused by the hysteresis and eddy current effects in the magnetic core. They can be estimated using the Steinmetz empirical formula:


$$P_{Iloss}=k\overset{\delta -1}{\underset{s}{f}}B^{\beta }V_{EE}$$ (4)

Among them, k, δ, and β are the core fitting coefficients provided by the manufacturer; $f_{s}$ is the switching frequency of the current flowing through the transformer; B is the saturation flux density of the transformer core; and $V_{EE}$ is the volume of the core.

(2) Station power consumption losses include the energy consumption of auxiliary equipment such as monitoring, lighting, and temperature control systems. In this paper, they are decomposed into two parts: fixed losses and temperature-dependent variable losses. The calculation formula is as follows:

$$P_{Selfloss}=P_{Fixed\_loss}+P_{Tem\_loss}\left(\Delta \theta \right)$$ (5)

Among them, $P_{Fixed\_loss}$ is fixed losses; $P_{Tem\_loss}$ is the temperature-dependent function; Δθ is the indoor-outdoor temperature difference.

#### 2.1.3. Generalization Guarantee of Loss Models in Dynamic Operating Scenarios.

To enhance the generalization ability of the loss models for battery packs, transformers, and station power consumption in dynamic operating scenarios such as sudden changes in load curves, fluctuations in ambient temperature, and battery aging, this paper introduces the following mechanisms in model construction:

(1) Parameter Adaptive Correction Mechanism: Key parameters such as the internal resistance of battery packs and the iron loss coefficient of transformers are not fixed. Instead, association functions between these parameters and operating states, including state of charge (SOC), state of health (SOH), ambient temperature, and load factor, are established through online identification or historical data fitting. For example, the battery internal resistance is modeled as a binary function of SOC and temperature, and the transformer iron loss coefficient is dynamically adjusted according to the actual operating current and frequency.
(2) Multi-operating Condition Data-driven Training: During the model training phase, historical data covering different seasons, various load ranges, and different charging/discharging operating modes is used for training. This enables the model to learn the nonlinear mapping relationships between losses and multiple factors, rather than relying on a single static curve.
(3) Real-time State Feedback Embedding: Real-time monitored state variables such as SOC, SOH, winding temperature, and ambient temperature are incorporated as part of the model inputs. This allows the loss calculation to be dynamically updated according to the current system state, thereby adapting to the fluctuations and gradual changes in actual operation.

Through the above designs, the loss models established in this paper not only possess theoretical physical interpretability but also can adapt to the complex dynamic scenarios faced by energy storage stations in actual scheduling, providing more realistic loss estimates for subsequent power forecasting.

### 2.2. Implicit Feature Extraction of Power Generation Capacity for Energy Storage Power Stations Based on Chaos-EEMD-PFBD

The power generation sequence of energy storage power stations is influenced by the complex interplay of multiple factors, including battery aging, equipment operating conditions, and environmental disturbances. Consequently, it exhibits strong nonlinearity and non-stationarity. Directly using the original sequence for prediction yields limited effectiveness. This section aims to systematically extract regular features that reflect the intrinsic system dynamics from seemingly chaotic univariate historical power data through a series of signal processing methods.

#### 2.2.1. Chaotic phase space reconstruction of power generation in energy storage power stations.

Considering that the power generation sequence of energy storage stations is not a simple linear superposition or periodic signal. Its fluctuations are profoundly influenced by a multitude of coupled nonlinear dynamic factors, including battery chemical characteristics, grid dispatching instructions, and random environmental disturbances, exhibiting inherent chaotic characteristics. Traditional time series decomposition methods such as Fourier transform, wavelet transform, or Seasonal-Trend Decomposition using LOESS (STL) typically assume that the data is stationary or has explicit periodic/trend components, making it difficult to effectively characterize such complex nonlinear dynamic behaviors.

Chaotic phase space reconstruction, based on Takens’ embedding theorem, maps a one-dimensional time series into a high-dimensional phase space using the delay coordinate method. This approach enables the reconstruction of a dynamic system trajectory that is topologically equivalent to the original system, thereby restoring implicit multidimensional state variables driving power variations from a single observed sequence, such as equivalent state of charge (SOC) changes, equipment efficiency, and external disturbances. This method is particularly well-suited for data like power generation sequences that exhibit non-stationary, nonlinear, and non-periodic characteristics, as it does not rely on a priori basis functions or periodicity assumptions. Instead, it directly mines the intrinsic dynamic laws from the data’s own structure, enabling more fundamental and robust extraction of complex features. Therefore, this paper selects chaotic phase space reconstruction to achieve the reconstruction of the chaotic phase space for the power generation sequence of energy storage stations.

For a historical power generation time series of an energy storage power station, it is first necessary to verify whether it exhibits chaotic characteristics. For chaotic time series, the most commonly used reconstruction method is the delay coordinate method. This approach samples the original sequence at fixed time intervals (delays) to construct *m*-dimensional phase-space vectors:


y(i)={y(i),y(i+τ),⋯,y(i+(m−1)τ)} (6)

Among them, {y(n)} is the point in the phase space at time *t*; *m* is the embedding dimension, which determines the dimensionality of the reconstructed space; and *τ* is the time delay, serving as the fundamental step size for reconstruction.

The selection of parameters *m* and τ is crucial. If *τ* is too small, adjacent coordinates become overly similar, leading to information redundancy; conversely, if *τ* is too large, the causal relationship between coordinates is lost. If *m* is too small, the dynamical system cannot be fully unfolded; whereas if *m* is too large, noise and computational complexity are introduced. This paper employs the C-C method, which can robustly estimate both *τ* and another key parameter—the time window $\tau_{w}$ —simultaneously, and then determines *m* using the relationship (m−1)τ.

The core of the C-C method lies in calculating the correlation integral statistic of the time series. The brief steps are as follows:

Step 1: Divide the time series of length *N* into *t* non-overlapping subsequences.

Step 2: For given embedding dimension *m*, time delay *τ*, and distance criterion r, calculate the correlation integral for each subsequence. It represents the proportion of point pairs in the phase space with a distance less than *r*, as shown in Equation (7).


$$\Phi \left(m,N,r,t\right)=\frac{\text{2}}{M\left(M-1\right)}\sum_{1\leq i\leq j\leq M}\psi \left(R-d_{ij}\right)$$ (7)

Among them, N is the size of the dataset, $d_{ij}$ is the ∞ -norm, ψ is the Heaviside function, and R is the maximum allowable distance between two points in phase space.

Step 3: Define the test statistic. It is essentially a measure of the volatility of the integral associated with different subsequences, as shown in equations (8) – (9).


$$S\left(m,N,r,t\right)=t^{-1}\sum_{s=1}^{t}\left[\Phi_{s}\left(m,\frac{N}{t},r,t\right)-\overset{m}{\underset{s}{\Phi }}\left(1,\frac{N}{t},r,t\right)\right]$$ (8)

Among them, r is used to evaluate the proximity between two points in phase space, $\Phi_{s}\left(m,\frac{N}{t},r,t\right)$ is the correlation integral value of the s -th subsequence under embedding dimension m, and $\overset{m}{\underset{s}{\Phi }}\left(1,\frac{N}{t},r,t\right)$ is the correlation integral of the s -th subsequence at position m=1.


$$S\left(m,r,t\right)=t^{-1}\sum_{s=1}^{t}\left[\Phi_{s}\left(m,r,t\right)-\overset{m}{\underset{s}{\Phi }}\left(1,r,t\right)\right]$$ (9)

Step 4: Select the first zero crossing point of $\bar{S}\left(t\right)$ or the first minimum value of $\Delta \bar{S}\left(t\right)$ as the delay τ, and take the minimum value of $S_{cor}\left(t\right)$ as the time window $\tau_{w}$. Then determine the m value and reconstruct the original one-dimensional power generation time series of the energy storage power station into an m -dimensional sequence.


$$\bar{S}\left(t\right)=\frac{\text{1}}{16}\sum_{m=2}^{5}\sum_{j=1}^{4}S\left(m,r_{j},t\right)$$ (10)

$$\Delta \bar{S}\left(t\right)=\frac{\text{1}}{\text{4}}\sum_{m=2}^{5}\left[\max\left\{S\left(m,r_{j},t\right)\right\}-\min\left\{S\left(m,r_{j},t\right)\right\}\right]$$ (11)

$$S_{cor}\left(t\right)=\Delta \bar{S}\left(t\right)+\left|\bar{S}\left(t\right)\right|$$ (12)

Through this process, this paper reconstructs the original one-dimensional power sequence into an *m*-dimensional phase space trajectory, where each dimension contains partial information about the original system’s state, laying the foundation for subsequent fine-scale decomposition.

#### 2.2.2. Phase Space Decomposition of Power Generation Time Series for Energy Storage Stations Based on EEMD.

Performing Ensemble Empirical Mode Decomposition (EEMD) on the power time series of energy storage stations in each dimension of the phase space can reduce the non-stationarity characteristics of the data in each dimension of the phase space, yielding a finite number of more regular Intrinsic Mode Functions (IMFs). This helps to minimize the impact of random fluctuations on the power prediction of energy storage stations. The specific steps are as follows:

(1) Assuming the sequence in phase space is z(t), the initial number of experiments is U, and the standard deviation coefficient of white noise amplitude is μ (including U=100 and W=0.1 parameters).
(2) Add the random Gaussian white noise $\zeta_{i}\left(t\right)$ to z(t) to obtain the noise-processed time series of the energy storage power plant’s generation output, i.e.,:

$$z_{i}\left(t\right)=z\left(t\right)+\mu \zeta_{i}\left(t\right)$$ (13)

(3) For the i -th EMD decomposition of $z_{i}\left(t\right)$, n IMF $u_{k,i}\left(t\right)$ s and 1 residual $r_{n,i}\left(t\right)$ are obtained.

$$u_{k,i}\left(t\right)=l_{j,i}\left(t\right),k=1,2,\cdots ,n$$ (14)

$$r_{n,i}\left(t\right)=r_{n-1,i}\left(t\right)-u_{n,i}\left(t\right)$$ (15)

The i -th IMF component that meets the standard ($l_{j,i}\left(t\right)$ is the power generation sequence of the power station to be decomposed, $l_{j,i}\left(t\right)=z_{j,i}\left(t\right)-\kappa_{j,i}\left(t\right)$ and $z_{j,i}\left(t\right)$ are related), with the upper and lower envelope mean $\kappa_{j,i}\left(t\right)$.

(4) If the relationship between i and U is i<U, then i=i+1 will be transformed (2); otherwise, proceed to (5).
(5) Calculate the EMD decomposition IMF and residual mean of U, which is the EEMD result.:

$$u_{k}\left(t\right)=\sum_{i=1}^{U}\left[u_{k,i}\left(t\right)/U\right],k=1,2,\cdots ,n$$ (16)

$$r_{n}\left(t\right)=\sum_{i=1}^{U}\left[r_{n,i}\left(t\right)/U\right]$$ (17)

#### 2.2.3. Extraction of Implicit Chaotic Features in Energy Storage Plant Power Output Based on PFBD.

This paper employs a frequency band division method based on the number of peak points (PFBD) to cluster and divide the Intrinsic Mode Function (IMF) components obtained from Ensemble Empirical Mode Decomposition (EEMD), aiming to provide a more precise and effective solution for power generation forecasting in energy storage stations.

After EEMD decomposition, the IMF components are arranged in descending order of frequency. However, their frequency boundaries are not strictly defined, and the number of IMFs obtained from decomposing sequences of different dimensions may vary. Directly using all components would result in excessively high feature dimensions and redundancy. PFBD divides the components based on their time-domain morphological characteristics (peak point density), which has a clear physical meaning. Specifically, the number of peak points directly reflects the frequency of signal oscillations, strongly correlated with the frequency level. This method avoids the adaptability issues of directly setting frequency thresholds for non-stationary signals and is computationally straightforward, facilitating the automated classification of a large number of IMFs into feature groups representing different time-scale dynamics (high-frequency random fluctuations and low-frequency trend variations).

Meanwhile, to avoid the arbitrariness of threshold setting, this paper adopts the following quantitative criteria to determine the threshold for dividing high-frequency and low-frequency components:

(1) Calculate the statistical distribution of the number of peak points across all IMF components and observe whether there are distinct bimodal patterns or breakpoints.
(2) Identify the point with the maximum gradient change in the natural logarithm of the peak point count as a candidate threshold point, which typically corresponds to the transition zone where signal components shift from “rapid oscillation” to “slow variation.”
(3) Incorporate a time-domain smoothness test after component reconstruction to ensure that the divided high-frequency components can effectively capture short-term disturbances, while the low-frequency components maintain smooth trends. Through the above data-driven quantitative analysis, a peak point count of 500 is ultimately determined as the division threshold. This value corresponds to a significant separation point in the statistical characteristics of the dataset, rather than being arbitrarily set based on empirical experience.

**Fig 2:**
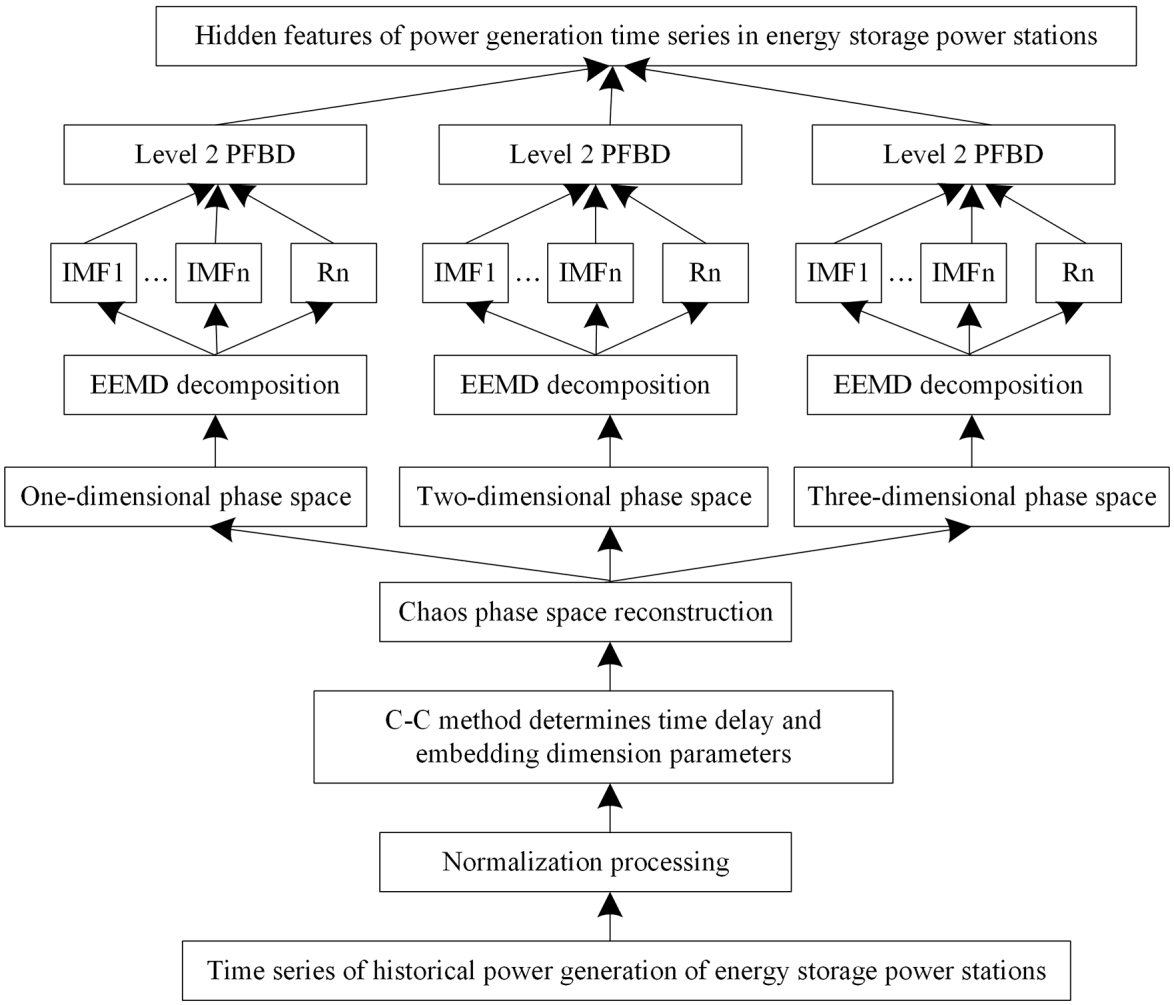
Flow chart for optimizing the extraction of hidden chaotic features in the power generation of energy storage power stations.

By integrating the above processes, the optimized extraction procedure for the implicit chaotic characteristics of power generation in energy storage stations is established, as illustrated in Fig 2.

The extraction procedure involves three main steps: First, the number of peak points for each Intrinsic Mode Function (IMF) component is calculated. Next, the threshold for dividing high-frequency and low-frequency components is determined by comprehensively considering the preservation of the overall trend of the original data, the separation of high-frequency components, and the enhancement of the stationarity of each component. Finally, the IMF components of the power generation time series within each phase-space dimension are reconstructed into aggregated high-frequency and low-frequency components.

By combining phase space reconstruction with EEMD and the PFBD method, the power data in each dimension of the phase space can be further decomposed and reconstructed, thereby excavating more implicit information for day-ahead power generation forecasting of energy storage stations.

### 2.3. CNN-LSTM-MLP-Based Day-Ahead Power Forecast Model for Energy Storage Power Stations

There are numerous challenges in predicting the power generation of energy storage stations recently. Firstly, the energy consumption characteristics of energy storage systems are complex. Battery pack losses are influenced by multiple factors, including the number of battery strings and the charging and discharging status. Additionally, transformer and plant power losses vary greatly, making accurate quantification difficult. Secondly, power generators exhibit chaotic characteristics with large fluctuations and are highly susceptible to internal and external random interference. Traditional methods are difficult to capture complex features and have poor prediction accuracy. Therefore, this article constructs a daytime power generation prediction model based on CNN-LSTM-MLP, which comprehensively considers energy consumption patterns and chaotic characteristics of power generation. The model input includes energy storage device losses and potential chaotic features of power generation. CNN extracts advanced features, identifies periodic changes and high and low frequency components, LSTM captures long-term temporal dependencies of power generation, MLP integrates the processing results, and finally outputs the predicted daily power generation through nonlinear transformation.

#### 2.3.1. Convolutional Neural Network (CNN).

**Fig 3:**
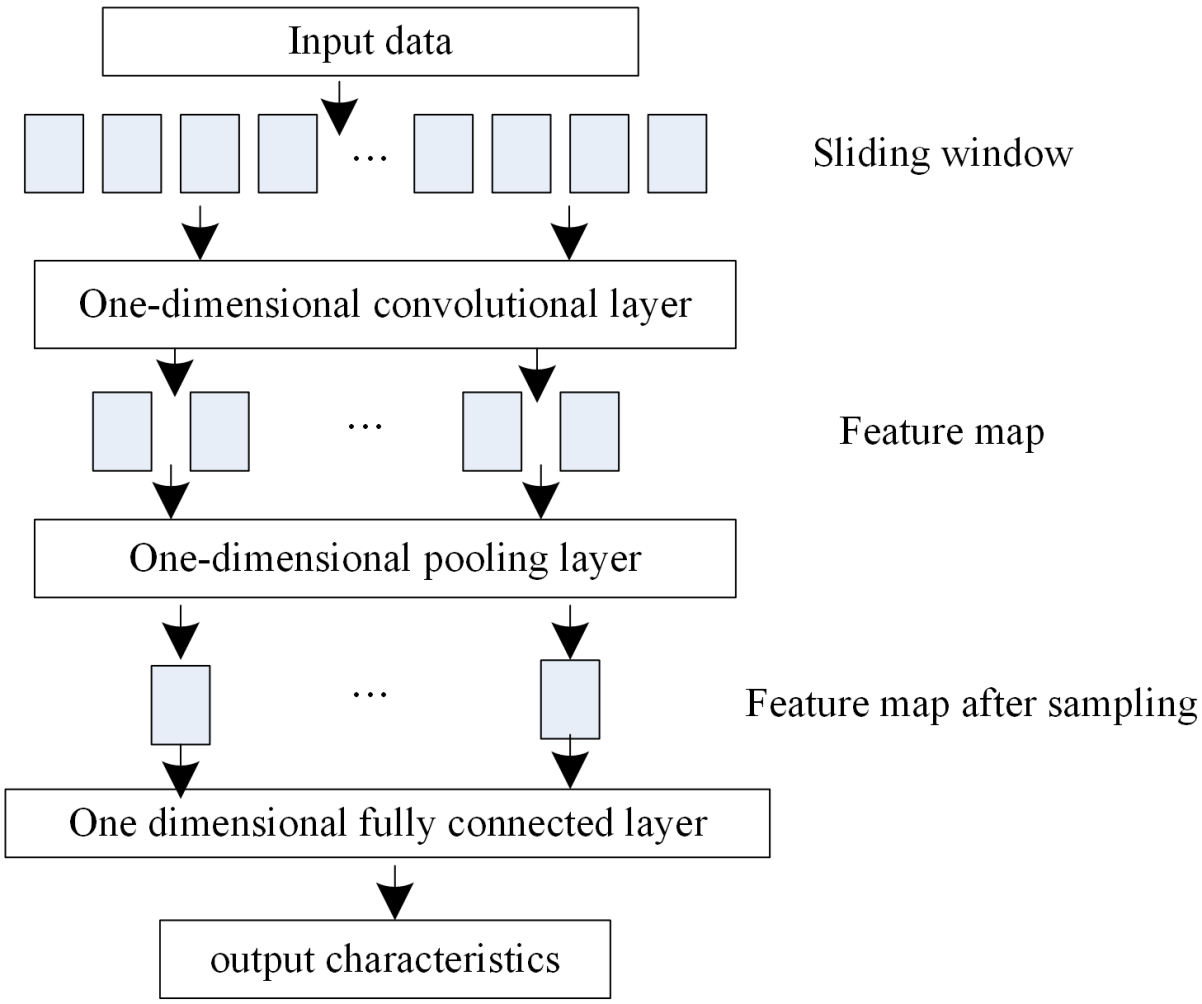
One-dimensional CNN network structure diagram.

CNN is a neural network designed specifically for processing data with known grid topology structures [17]. Through convolution operations, it consists of input, convolution, pooling, fully connected, and output layers. Due to the fact that the input of the model is mostly time series data, this article chooses a one-dimensional CNN, whose structure is shown in Fig 3. Eliminate dimension conversion between input and output. Its one-dimensional convolution kernel adopts unidirectional sliding with time step intervals, which is more suitable for time series convolution processing.

#### 2.3.2. Long Short-Term Memory (LSTM) Network.

LSTM is time sensitive and can capture sequence data features. Due to the limitations of CNN in extracting temporal features, this paper uses LSTM, which relies on three gates to iteratively update the state and dependencies, achieving efficient information control and transmission.

Considering improved versions such as FE-S-BiLSTM (Forward Encoding-Selective Bidirectional LSTM), CNN-EFC-BiLSTM (Convolutional-Enhanced Feature Compression Bidirectional LSTM), or LSTM-Reduction, which typically enhance performance by introducing more complex gating mechanisms, bidirectional structures, or feature compression, these modifications also significantly increase model parameter counts and computational complexity. For the day-ahead forecasting task in this paper, where temporal dependencies primarily manifest as forward causality (future power depends on past states), the advantages of bidirectional LSTMs are less pronounced. Additionally, within the CNN-LSTM-MLP hybrid architecture proposed herein, the CNN is already responsible for extracting local spatiotemporal features, while the MLP handles high-order nonlinear mappings. The classical LSTM, in this framework, focuses on capturing medium- to long-term temporal dependencies, and its structure adequately meets the requirements. Global optimization of all model parameters, including those of the LSTM, using an adaptive genetic algorithm can partially compensate for potential limitations in representational capacity inherent in simpler structures while achieving a better balance between model complexity and overfitting risk. Therefore, this paper selects the classical LSTM unit as the temporal feature extractor. LSTM processes input data through the following formula:


$$\left\{ \begin{array}{c} f_{t}=\sigma \left(W_{xf}\cdot x_{t}+W_{hf}\cdot h_{t-1}+b_{f}\right)\\ i_{t}=\sigma \left(W_{xi}\cdot x_{t}+W_{hi}\cdot h_{t-1}+b_{i}\right)\\ {\tilde{C}}_{t}=\tanh\left(W_{xc}\cdot x_{t}+W_{hc}\cdot h_{t-1}+b_{c}\right)\\ C_{t}=f_{t}\cdot C_{t-1}+i_{t}\cdot {\tilde{C}}_{t} \end{array}\right.$$ (18)

$f_{t}$ is the activation value of the forget gate (a vector between 0 and 1), which determines the proportion of long-term memory $C_{t-1}$ retained in the current step; σ is the sigmoid function, compressing the input to 0–1. $W_{xf}$ is the weight matrix of the forget gate input, $x_{t}$ is the current step input vector, $h_{t-1}$ is the hidden state from the previous moment, $W_{hf}$ is its weight matrix for the forget gate, and $b_{f}$ is the forget gate bias vector. $i_{t}$ is the activation value of the input gate, which determines the current amount of input information to be merged into the long-term memory; $W_{xi}$. $W_{hi}$ is the weight matrix from $x_{t}$ and $h_{t-1}$ to the input gate, and $b_{i}$ is the bias vector. ${\tilde{C}}_{t}$ is the current candidate storage unit state (hyperbolic tangent function value), $W_{xc}$ and $W_{hc}$ are the weight matrices from $x_{t}$, $h_{t-1}$ to ${\tilde{C}}_{t}$, $b_{c}$ is the bias vector, and $C_{t}$ is the current storage unit state.

#### 2.3.3 Multi-Layer Perceptron (MLP).

The Multi Layer Perceptron (MLP), as a feedforward neural network model [18], relies on the weight matrix and activation function to transform the input data multiple times in order to learn nonlinear patterns in the data.

When calculating the output of each layer, the output of the previous layer’s neurons and their connection weights are first weighted and summed, and then a nonlinear transformation is implemented through an activation function to enable MLP to capture complex nonlinear relationships in the data. Let the output of the i -th layer’s neurons be as follows:


$$Y_{i}=\lambda \left(\sum_{j}\omega_{ij}x_{j}+b_{j}\right)$$ (19)

$x_{j}$ is the output of the i−1 -th neuron, $\omega_{ij}$ is the connection weight, $b_{j}$ is the bias, and λ is the activation function.

MLP learns input-output mappings through backpropagation and gradient descent algorithms, offering advantages of simple structure and efficient training. This paper feeds CNN and LSTM processing results into the MLP to achieve final daily power generation forecasting for energy storage power stations.

#### 2.3.4 Model Regularization and Overfitting Mitigation Strategies.

Given the high dimensionality of input features extracted through EEMD-PFBD and the loss model, coupled with a relatively limited training data period (23 months), the CNN-LSTM-MLP hybrid architecture faces potential risks of overfitting. To enhance the model’s generalization capability, this paper employs a comprehensive set of strategies during training, as follows:

(1) **Dropout Regularization**: Dropout layers are introduced in the fully connected layers of the CNN, between LSTM layers, and within the hidden layers of the MLP. During training, a portion of neurons are randomly dropped out to disrupt complex co-adaptation relationships among neurons, forcing the network to learn more robust features.
(2) **Early Stopping**: Model performance is monitored on the validation set. Training is terminated prematurely when the validation loss fails to decrease over consecutive training epochs, preventing the model from overfitting to noise in the training set.
(3) **L2 Weight Regularization**: L2 regularization terms are added to the weights of the CNN, LSTM, and MLP layers to penalize excessively large weight values. This encourages the model to remain concise and avoids excessive sensitivity to incidental noise in the training data.
(4) **Implicit Regularization via Adaptive Genetic Algorithm**: The adaptive genetic algorithm, with its balanced mechanism of global search and local fine-tuning during parameter optimization, helps prevent the optimization process from converging to local optima that fit the training data well but generalize poorly. This indirectly enhances the model’s generalization performance.

Through the combination of these strategies, this paper effectively controls model complexity with limited data, enhancing feature learning capabilities on the training set while ensuring prediction stability on unseen test data.

### 2.4 Model Parameter Optimization Based on Adaptive Genetic Algorithms

Genetic algorithm simulates natural survival of the fittest, using selection, hybridization, and mutation to find the optimal solution. The output prediction solution space of energy storage power plants is complex and influenced by multiple factors, making parameter optimization challenging [19–20]. The adaptive genetic algorithm adjusts the crossover $P_{c}$ and mutation $P_{m}$ probabilities based on fitness, and is more conducive to global search when the fitness is low. The adjustment formula is as follows:


$$P_{c}=\left\{ \begin{array}{c} P_{c2},e^{\prime }<e_{avg}\\ \frac{P_{c1}\left(e_{\max}-e^{\prime }\right)}{e_{\max}-e_{avg}},e^{\prime }\geq e_{avg} \end{array}\right.$$ (20)

$$P_{m}=\left\{ \begin{array}{c} P_{m2},e<e_{avg}\\ \frac{P_{m1}\left(e_{\max}-e^{\prime }\right)}{e_{\max}-e_{avg}},e\geq e_{avg} \end{array}\right.$$ (21)

$e_{\max}$ is the maximum fitness of the population, $e_{avg}$ is the average fitness of each generation, $e^{\prime }$ is the larger fitness of the two hybrid individuals, and e is the fitness of the individual to be mutated, $P_{c1}$、 $P_{c2}$、 $P_{m1}$、 $P_{m2}$ ∈(0,1) [21–23].

The parameter optimization of the daily power generation prediction model for energy storage power stations is as follows:

(1) Extract weight and bias parameters from the energy storage power plant daily power generation forecast model using an adaptive genetic algorithm. These serve as genetic genes, encoded using the real number coding method for reduced computation time and enhanced accuracy.
(2) Randomly generate an initial population, where all genes within the population fall within the range (−1, 1).
(3) Compare fitness values across individuals. The fitness function uses the reciprocal of the total network error, calculated as:

$$E=\frac{\text{1}}{\sum_{j=1}^{J}\sum_{i=1}^{I}\;{\left[X_{kj}\left(n\right)-{X^{\prime }}_{kj}\left(n\right)\right]}^{2}}$$ (22)

Where: $X_{kj}\left(n\right)$ and $\overset{\prime }{\underset{kj}{X}}\left(n\right)$ represent the model’s expected output and actual output, respectively.

(4) According to the fitness values in the previous step, select, crossover, and mutate individuals, and calculate the crossover and mutation probabilities using equations (20) and (21).
(5) Repeat the above two steps to generate a new generation of the population until the number $T_{\max}$ of iterations requirement is met.
(6) Select the best individual with the highest fitness from the $T_{\max}$ generation group and input its parameters into the daily power generation prediction model of the energy storage power plant.
(7) Train the model with optimal parameters, input test samples, and predict the daily power generation of the energy storage power station.

To balance the convergence speed and solution quality of the genetic algorithm, this paper conducts a systematic sensitivity analysis on key parameters (population size and mutation probability). The specific experimental setup is as follows: The initial crossover probability (0.3) and other model parameters are fixed, while different population sizes (80, 120, 160) and initial mutation probabilities (0.3, 0.4, 0.5) are tested to assess their impacts on algorithm performance. The evaluation metrics include the number of iterations required for convergence (to measure convergence speed) and the mean squared error (MSE) of the optimized model on the test set (to measure solution quality). The results are shown in Table 1.

**Table 1:** Results of the Sensitivity Analysis on Genetic Algorithm Parameters.

Parameter Combination	Convergence Iterations	Final MSE (MW²)	Convergence Stability
Population = 80, Mutation = 0.3	342	6.85	High
Population = 80, Mutation = 0.4	298	6.92	Medium
Population = 80, Mutation = 0.5	265	7.21	Low
Population = 120, Mutation = 0.3	376	6.55	High
Population = 120, Mutation = 0.4	326	6.41	High
Population = 120, Mutation = 0.5	288	6.78	Medium
Population = 160, Mutation = 0.3	410	6.48	High
Population = 160, Mutation = 0.4	359	6.45	High
Population = 160, Mutation = 0.5	312	6.63	Medium

As can be seen from Table 1, the population size has a significant impact on algorithm performance: As it increases, the algorithm’s ability to find better solutions improves, with the final MSE decreasing from 6.85 to 6.45. However, the number of iterations required for convergence also increases significantly, from 342 to 410. When the population size is 120, a good balance is achieved between solution quality and convergence speed. Regarding the mutation probability, a higher value (0.5) can accelerate early convergence and reduce the number of iterations, but it may lead to a decline in solution quality and an increase in MSE. This is because premature over-exploration disrupts the excellent genes of individuals. When the mutation probability is 0.4, it can better protect excellent genes while maintaining good exploration capabilities. Through multiple independent repeated experiments to evaluate convergence stability, it is found that the combination of a population size of 120 and a mutation probability of 0.4 exhibits the highest convergence stability, with low dependence on the random initial population and good robustness. Based on the above analysis, the parameter configuration of a population size of 120 and an initial mutation probability of 0.4 is finally selected. This configuration can ensure solution quality (MSE = 6.41), has a relatively fast convergence speed (326 iterations), and features a stable convergence process.

## 3. Experimental analysis

**Fig 4:**
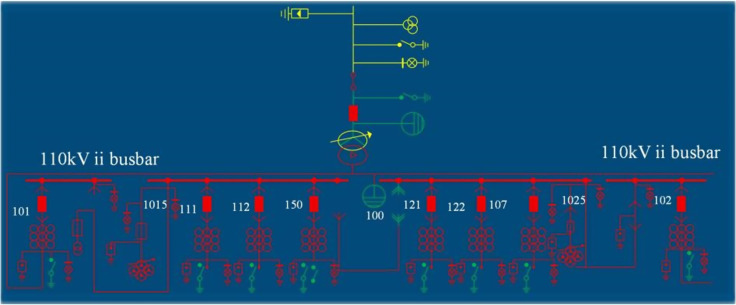
Experimental Subject.

An electrochemical energy storage power station with a capacity of 10 MW/20 MW·h in a certain region was selected as the experimental subject (the system structure is shown in Fig 4). This power station primarily undertakes tasks such as peak shaving and valley filling on the grid side and smoothing out fluctuations in new energy sources. To comprehensively collect experimental data, the power station is equipped with a complete monitoring system:

(1) Power and Energy Monitoring: Smart meters with a precision class of 0.5S are installed at the 10 kV grid connection point and at the outlet of each energy storage unit. These meters are used to simultaneously measure and record active power, reactive power, and energy data, with configurable sampling intervals. In this experiment, 5-minute average power values are adopted as the basic data points.
(2) Battery System Monitoring: Each battery cluster is equipped with a Battery Management System (BMS) that continuously monitors and uploads key parameters such as battery voltage, current, temperature, State of Charge (SOC), and State of Health (SOH) to the power station-level monitoring platform in real time.
(3) Environmental and Auxiliary System Monitoring: Temperature and humidity sensors are deployed within the power station to monitor environmental conditions in real time. The station’s auxiliary power consumption is separately metered using independent energy measurement devices.

The power station is configured with three energy storage units, each with a capacity of 3.45 MW/6.73 MW·h, connected in parallel to the 10 kV switchgear busbar. Each energy storage unit consists of two energy storage subsystems, with each subsystem containing a Power Conversion System (PCS) rated at 1.725 MW and a lithium iron phosphate battery pack rated at 3.36 MW·h. There are a total of six battery packs across the three energy storage units, with each battery pack containing 10 battery clusters and 60 battery stacks. Among these, 40 battery stacks are each composed of 64 individual battery cells connected in series, while the remaining 20 battery stacks are each composed of 60 individual battery cells connected in series. The rated capacity of each battery cell is 280 Ah.

The data collection and processing procedures are as follows: Historical power generation data (referring to the net power actually fed into the grid) from January 2022 to December 2023, spanning a total of 24 months, were collected from this energy storage power station to construct the experimental dataset. After preprocessing the raw data by removing obvious outliers (using the 3σ principle) and filling in missing values (using linear interpolation between adjacent time points), the first 23 months of data were selected as training samples, and the last month’s data were used as test samples to validate the model’s generalization capability.

The group size is 120, with 500 iterations, an initial crossover probability of 0.3, and a mutation probability of 0.4. The prediction model was developed within a Python deep learning framework. Subsequently, its performance advantage in multi-day-ahead power generation forecasting for energy storage stations was verified.

**Fig 5:**
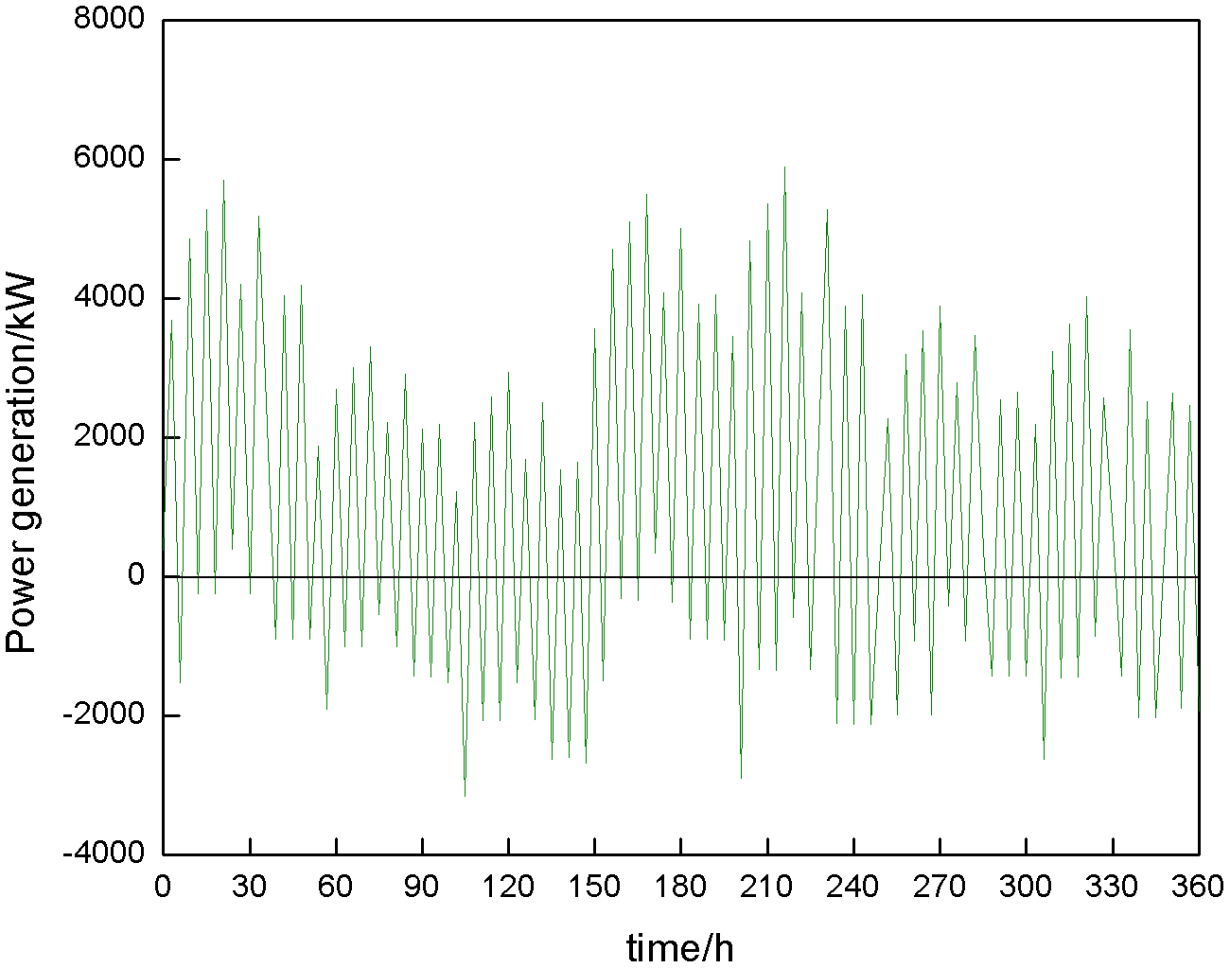
Time series of historical power generation of energy storage power station.

**Fig 6:**
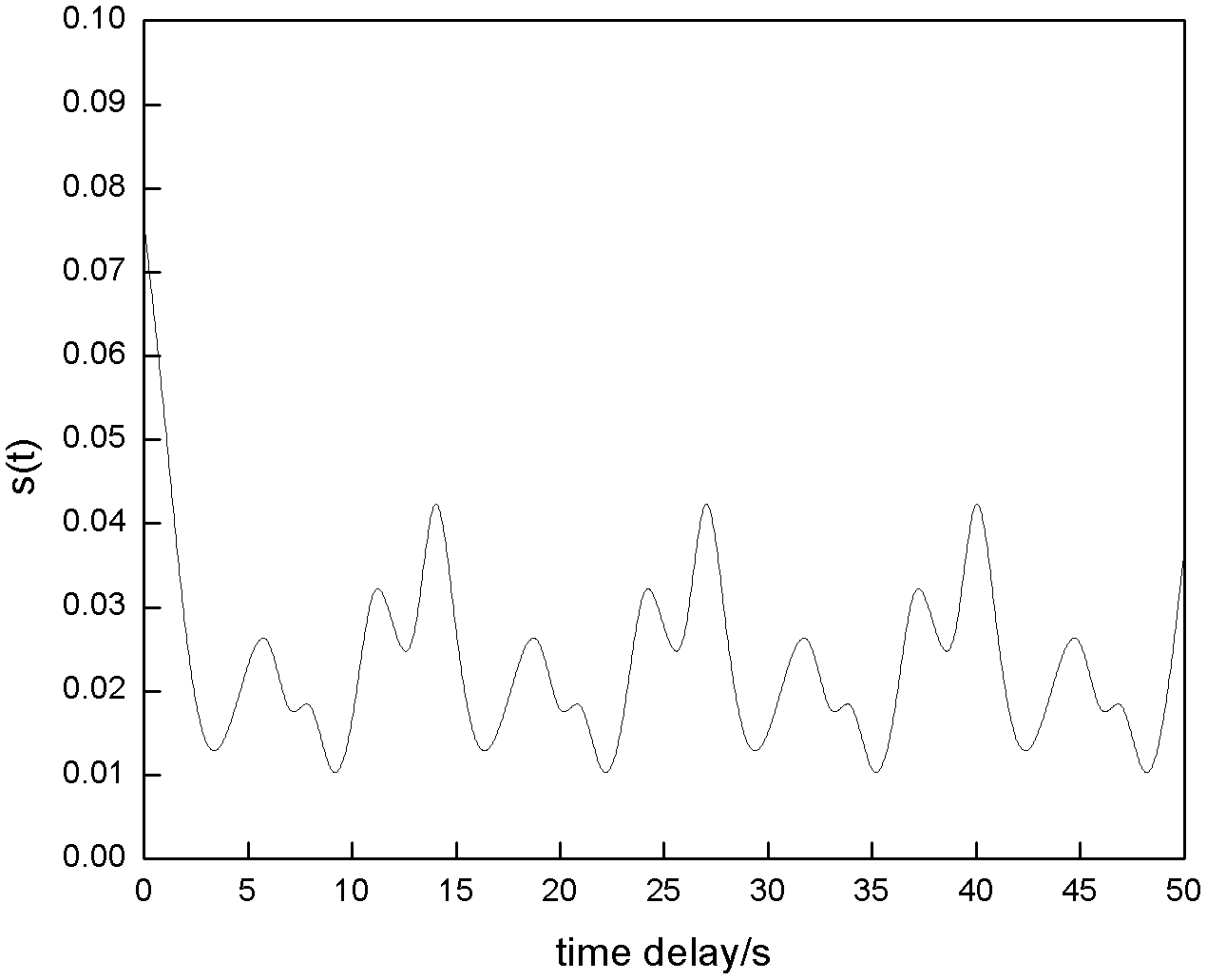
$\Delta \bar{S}\left(t\right)$ Curve.

**Fig 7:**
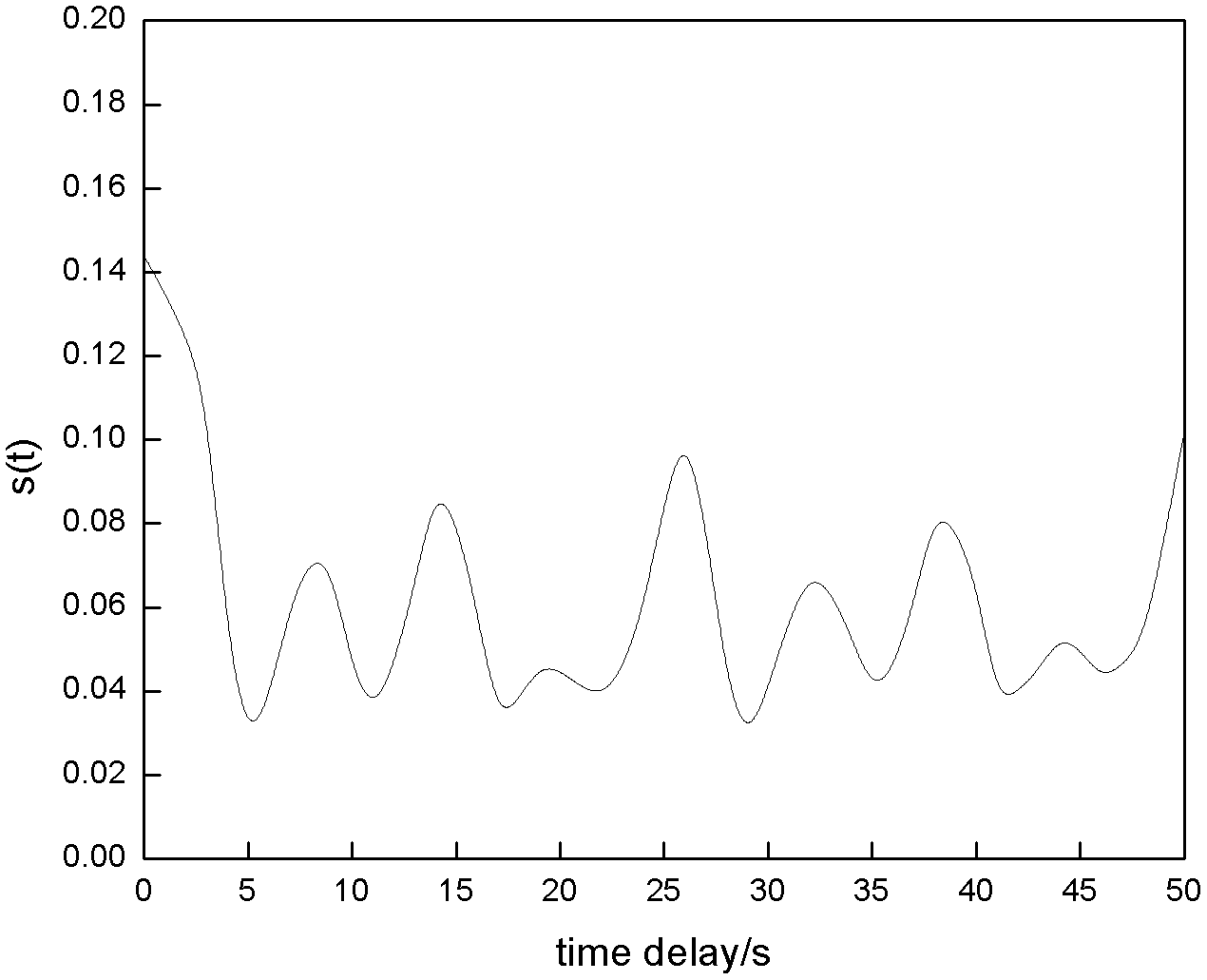
$S_{cor}\left(t\right)$ Curve.

Fig 5 shows the power generation sequence of the energy storage power station. The chaotic phase space is reconstructed using an algorithm, and the time delay relationship between $\Delta \bar{S}\left(t\right)$ and $S_{cor}\left(t\right)$ is analyzed to determine the optimal parameters. The results are shown in Figs 6 and 7.

Analysis of Figs 6 and 7 shows that the first minimum point of $\Delta \bar{S}\left(t\right)$ corresponds to a time delay of 4 seconds, and the optimal time delay τ for reconstructing the historical power generation sequence of the energy storage power station is determined to be 4; The first minimum point of $S_{cor}\left(t\right)$ corresponds to a time delay of 6 seconds, and the optimal embedding dimension m is calculated to be 3, which can reconstruct the historical power generation sequence.

After reconstructing the original historical power generation sequence of the energy storage power station into phase space, EEMD decomposition is performed on the power sequence of each dimension, and the “findpeaks” function is called to determine the number of peak points for each component. PFBD segmentation is performed according to the peak distribution pattern, with a threshold of 500. Components below this value are classified as low frequency, while those below this value are classified as high frequency. The potential features of the original sequence are fully extracted, and the number of peak points for each dimension phase space component is shown in Table 2.

**Table 2:** Calculation results of peak points for different decomposition components in various dimensional phase spaces.

Weight		One-dimensional phase space	Two-dimensional phase space	Three-dimensional phase space
High-frequency component	U1	4610	4624	4615
	U2	1820	1865	1849
	U3	910	925	936
Low-frequency component	U4	281	283	267
	U5	102	114	111
	U6	65	64	62
	U7	22	21	21
	U8	15	15	15
	U9	8	8	8
	U10	2	2	2
	U11	2	2	2
	R1	1	1	1

Table 2 shows that the processed energy storage power generation sequence obtains stable high and low frequency components, which accurately present the dynamic response of the power plant at different time scales, indirectly reflecting the internal mechanism regulated by multiple external factors. In each dimension of phase space, the low-frequency components are reconstructed by superimposing U4-U11 decomposed by EEMD and residual R1, reflecting the slow power variation trend and long-term fluctuations; Overlay U1-U3 to reconstruct high-frequency components and capture power fast short-term interference and high-frequency oscillations.

**Fig 8:**
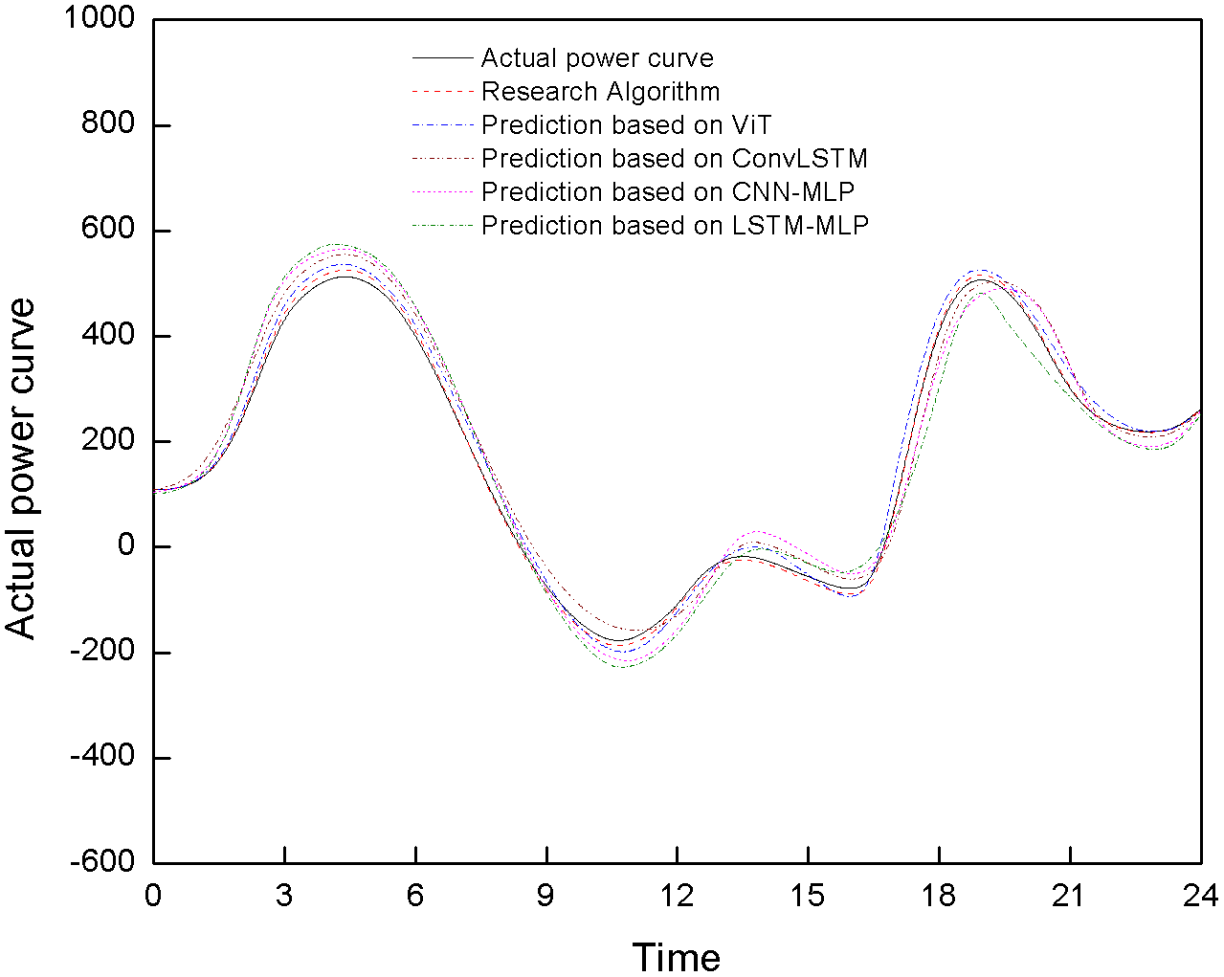
Comparison results of power generation forecast curves for energy storage power stations under different algorithms.

To verify the effectiveness of the algorithm, multiple algorithms were used to test the 24-hour power generation of the energy storage power station. Fig 8 shows the comparison of the predicted curves.

The analysis of Fig 8 shows that the proposed algorithm performs excellently in the daily power generation prediction of energy storage power stations. The predicted curve is highly consistent with the actual power curve, accurately capturing power trends with minimal differences, highlighting its reliability and accuracy in power prediction. In contrast, algorithms based on CNN-MLP have insufficient ability to mine deep temporal features, resulting in significant prediction bias during high-power fluctuations and inability to thoroughly analyze the complex temporal variation patterns of power output in energy storage power plants. The algorithm based on LSTM-MLP has advantages in processing temporal features and can effectively capture the temporal patterns of power generation data. However, power generation is affected by various local factors such as load, renewable energy output, and battery SOC, which have complex spatial correlations and distribution characteristics. This algorithm lacks spatial modeling capabilities and cannot fully extract and utilize spatial information, resulting in large errors in predicting power generation and limiting accuracy improvement. The algorithm based on ViT has a certain ability in local feature extraction, which can capture some local features, but the time correlation capture is insufficient, and the continuity and correlation of the power data time dimension are not fully grasped. The overall prediction curve deviates from reality, but the deviation is smaller compared to CNN-MLP and LSTM-MLP algorithms. The algorithm based on ConvLSTM can theoretically handle spatiotemporal features, but in reality, it cannot accurately predict power mutations due to limitations in spatiotemporal fusion and dynamic modeling.

Table 3 presents the performance differences of the above algorithms in terms of MSE, MAE, and R²metrics.

**Table 3:** Comparison of performance of different prediction methods.

Method	MSE	MAE	R^2^
CNN-MLP	10.72	9.88	0.817
LSTM-MLP	10.89	9.78	0.832
ViT	7.95	8.75	0.855
ConvLSTM	7.58	8.88	0.861
Research method	6.41	7.68	0.898

Table 3 shows that the algorithm performs outstandingly in the 24-hour power generation prediction of energy storage power stations, with MSE, MAE, and R²all achieving the best results. MSE is 6.41, and the prediction has the smallest squared deviation and the highest accuracy; MAE is 7.68, with the smallest average absolute prediction error and stronger robustness; The R² reaches 0.898, and the model fitting quality is excellent, fully verifying its advantages in daily power generation forecasting.

In addition, the algorithm is also used to predict the power generation of energy storage stations on December 30, 2023. The prediction results at different time points are shown in Table 4.

**Table 4:** Analysis of day-ahead forecast results of power generation in energy storage power stations.

Time	Predicted power/MW	Actual power/MW	Absolute Error/ MW	Relative Error/ %
0:00	1.2	1.10	0.10	9.09
2:00	1.0	0.95	0.05	5.26
4:00	0.9	0.88	0.02	2.27
6:00	1.5	1.45	0.05	3.45
8:00	1.7	1.72	0.02	1.16
10:00	3.8	3.67	0.13	3.54
12:00	4.2	4.16	0.04	0.96
14:00	4.0	3.94	0.06	1.52
16:00	3.5	3.42	0.08	2.34
18:00	2.8	2.69	0.11	4.09
20:00	3.0	2.91	0.09	3.09
22:00	2.5	2.43	0.07	2.88
24:00	2.0	1.95	0.05	2.56

Note: The relative error calculation result is rounded to two decimal places.

Table 4 analysis shows that there is a significant difference in the power generation of energy storage stations at different times: during the low load period from 0:00–6:00, the power generation is low and fluctuates moderately; 8: From 00:00–16:00, due to the increase in renewable energy production and changes in electricity demand, the power generation gradually increases and maintains a high level; 18: After the evening rush hour from 00:00–24:00, the power generation gradually decreases again. Comparing the predicted and actual power, the absolute error at each time point is 0.02–0.15 megawatts, and the relative error is mostly within 5%. This indicates that the predicted values of the research algorithm are highly consistent with the actual situation, accurately capturing the trend of electricity in various periods. In scenarios such as low load, peak renewable energy output, and evening rush hour, the performance of the algorithm is robust, effectively verifying its outstanding performance in the prediction of daily power generation in energy storage power plants.

This method extracts implicit features from historical data through chaotic phase – space reconstruction. The adaptability of this method to power – generation mode mutations caused by grid disturbances or equipment failures depends on the frequency and representation degree of mutation events in historical data. To verify this adaptability, the following test was designed: In the test data of December 2023, two typical mutation patterns were artificially injected: (1) Simulate the instantaneous power drop caused by grid voltage sag (lasting for 10 minutes with an amplitude of 30% of the rated power); (2) Simulate the step – like power decline caused by PCS failure (lasting for 2 hours with an amplitude of 50% of the rated power). The data containing mutations was input into the trained model, and its prediction performance was observed. The results are shown in Table 5.

**Table 5:** Prediction Performance under Mutation Scenarios.

Mutation Type	Mutation Period	Prediction MSE (MW²)	Prediction MAE (MW)	Mutation Point Detection Delay
Voltage Sag	12-15 14:00-14:10	15.23	10.45	5 minutes
PCS Failure	12-20 10:00-12:00	28.67	15.32	30 minutes
No Mutation	Entire Test Set	6.41	7.68	Not Applicable

As can be seen from Table 5, for short – term instantaneous mutations such as voltage sag, although the model has a certain tracking ability, there is a delay of about 5 minutes and the error increases significantly. This indicates that it can learn some similar patterns from historical fluctuations but has poor prediction ability for the exact occurrence time. For long – term and large – amplitude mutations such as PCS failure, the model takes about 30 minutes to adjust the prediction to a new stable level, with extremely large errors during this period. The reason is that such events rarely appear in the training data, and the model fails to fully learn their patterns. This method is essentially a statistical learning method based on historical patterns, and its prediction ability is limited by the range of operating states covered by the training data. It has a certain adaptability to small disturbances that have occurred or are similar in history, but for new and large – scale fault patterns, additional fault detection mechanisms and model online update strategies are required. To enhance the adaptability to mutations, future improvement directions include introducing more simulated data of fault and disturbance scenarios into the training data, constructing a mutation detection module to switch to a short – term rolling prediction mode based on real – time data when anomalies are detected, and adopting an online learning mechanism to enable the model to continuously update using the latest data.

The computational cost of the method proposed in this paper mainly comes from two parts: daily feature extraction (Chaos-EEMD-PFBD) and prediction using the CNN-LSTM-MLP model optimized by an adaptive genetic algorithm. To evaluate its feasibility for daily operation in actual energy management systems, a complete runtime test was conducted on a standard server (Intel Xeon Gold 6248R CPU, 128GB RAM, NVIDIA Tesla V100 GPU). The results are shown in Table 6.

**Table 6:** Analysis of Computational Time for Each Stage.

Computational Stage	Single-Run Time	Parallelism	Remarks
Chaotic Phase-Space Reconstruction	2.3 seconds	Low	Depends on complete daily data
EEMD-PFBD Feature Extraction	18.7 seconds	High (independent for each dimension)	Main time-consuming stage
CNN-LSTM-MLP Forward Prediction	0.8 seconds	High	Significant GPU acceleration
Adaptive Genetic Algorithm Optimization (Training Phase)	Approximately 4.2 hours	Medium (population-based parallelism)	Only required periodically
Entire Process (excluding training)	Approximately 22 seconds	Partial parallelism	Daily prediction cost

As can be seen from Table 6, in terms of daily prediction feasibility, excluding the time-consuming model training/optimization phase, completing a day-ahead prediction takes only about 22 seconds. Given that predictions are usually carried out at a fixed time each day (such as midnight), there is an ample time window, making this time consumption entirely feasible in practical engineering. Regarding training cost and update frequency, model training (including genetic algorithm optimization) takes about 4.2 hours, but it does not need to be performed daily. In actual deployment, retraining can be carried out on a weekly or monthly basis, or triggered when a decline in model performance is detected. Daily predictions only use the pre-trained model for forward computation. In terms of resource requirements, the use of a GPU significantly accelerates the inference process of the CNN-LSTM-MLP model. For edge devices without a GPU, the prediction time will increase to about 5 seconds when only using a CPU, which is still within an acceptable range. To enhance engineering practicality, model distillation techniques can be further adopted to compress the complex model into a lightweight version for deployment on resource-constrained devices, parallel computing in the feature extraction stage can be implemented to shorten the time, and incremental learning algorithms can be developed to avoid complete long-duration training each time. In conclusion, from the perspective of computational resources, this method has already demonstrated the feasibility of daily operation in actual energy management systems. The key lies in decoupling the time-consuming training process from the daily prediction process and reasonably arranging their execution frequencies.

## 4. Discussion

### 4.1. Physical meaning and engineering interpretation of forecasting errors

Experimental results show that for an energy storage station with a rated power of 10 MW, the mean squared error (MSE) of the day-ahead forecasting using the proposed method is 6.41 MW², and the mean absolute error (MAE) is 7.68 MW. These statistical metrics have clear physical interpretations: the square root of MSE (RMSE ≈ 2.53 MW) represents the standard deviation of the forecasting error sequence, reflecting the dispersion of errors around zero; while MAE directly indicates the average magnitude of absolute forecasting errors, meaning that, on average, each forecast deviates from the actual value by approximately 7.68 MW. To further understand its engineering impact, MAE can be converted into a percentage error relative to the rated power, approximately 7.68%. This error level implies that, on a typical operating day, there exists an average-magnitude systematic deviation between the forecasted and actual power curves.

### 4.2. Matching analysis with operational tolerance levels of energy storage systems

To evaluate the acceptability of forecasting errors, it is essential to consider the specific application scenarios of energy storage stations in the power grid and their inherent operational tolerance capabilities. The requirements for power accuracy vary significantly across different tasks of energy storage systems:

(1) **High-precision application scenarios (e.g., primary frequency regulation, automatic generation control (AGC))**: These typically require tracking errors of power commands to be strictly controlled within 1%−5% of the rated power to ensure rapid and precise regulation of grid frequency. The current average relative error of approximately 7.68% using the proposed method may exceed the allowable tolerance range for direct closed-loop control in such scenarios.
(2) **Energy-oriented application scenarios (e.g., peak shaving, energy time-shifting)**: The core objective is to achieve energy transfer across time scales, with relatively relaxed requirements for instantaneous accuracy of single-point power and greater emphasis on the balance of total energy over the day. In such scenarios, the day-ahead forecasting curve provided by this study (R² = 0.898) can accurately depict the overall trend of power variations, offering valuable reference for formulating charging and discharging plans for energy storage systems. The system can accommodate forecasting errors of this magnitude by reserving appropriate adjustment margins or incorporating ultra-short-term rolling corrections.
(3) **Integration with the inherent regulation capabilities of energy storage**: It should be noted that energy storage stations inherently possess rapid power response capabilities ranging from milliseconds to seconds. Therefore, the day-ahead forecasting results of this study are more suitable as feedforward inputs for upper-level energy management strategies (e.g., model predictive control (MPC)) or as benchmarks for plan formulation. During actual operation, the rapid regulation characteristics of energy storage can be utilized to dynamically compensate for deviations between day-ahead forecasts and real-time states, thereby meeting higher-precision operational requirements while fully leveraging the planning and guidance value of day-ahead forecasts.

In summary, the forecasting accuracy of the proposed method in this study holds high practical value in scenarios such as energy management and plan formulation, providing reliable decision-making bases for dispatchers. For scenarios with extremely high power control accuracy requirements, the forecasting outputs of this study can serve as foundational references but need to be used in conjunction with real-time closed-loop control strategies. Future research can further focus on reducing extreme errors to broaden the direct applicability of this method across a wider range of regulation scenarios.

## 5. Conclusion

In response to the challenges of difficulty in loss quantification and insufficient extraction of chaotic features in the day-ahead forecasting of power generation for energy storage power stations, this paper proposes a hybrid prediction method that integrates a refined physical model, chaotic data mining, and a deep learning optimization framework. Through empirical research, it has been demonstrated that the constructed refined loss model can effectively quantify energy dissipation and enhance the physical interpretability and fundamental accuracy of net power prediction. The proposed feature extraction process can uncover the multi-scale dynamic characteristics implicit in univariate historical power sequences. The “CNN-LSTM-MLP hybrid model architecture combined with Adaptive Genetic Algorithm (AGA) optimization” can synergistically capture various relationships of energy storage power, and its prediction performance significantly outperforms multiple benchmark and comparison models. A comprehensive evaluation indicates that this method has advantages in prediction accuracy and an acceptable daily prediction computational cost, but it also faces limitations such as limited adaptability to unseen historical mutations and high model complexity.

Although the method proposed in this study exhibits superiority in experimental scenarios, there are still several limitations and challenges that may arise in practical applications, which need to be objectively examined. Firstly, the core feature extraction relies on historical power data, resulting in a decreased prediction capability for power generation mode mutations not present in the training data. Essentially, it is an “extrapolation” approach that struggles to predict entirely new operating states. Secondly, the proposed framework integrates multiple components, leading to a complex model structure with numerous parameters. The AGA optimization process is time-consuming, resulting in high costs during frequent updates, and the deployment of complex models on edge computing devices is challenging. Thirdly, the physical model is a simplified representation, and the acquisition of key parameters depends on manufacturer data or extensive experiments, which may be difficult to obtain accurately in old power stations, affecting input accuracy and the final prediction results. Additionally, the method has been validated in a specific power station, and when applied to energy storage power stations of different types, scales, or scheduling strategies, some parameters, model structures, and hyperparameters need to be readjusted or retrained, and its universality requires further verification in different cases.

Based on the above research, future work can follow the following guidelines. Firstly, enhance the model’s online adaptability and mutation response capability by researching hybrid prediction frameworks that incorporate real-time anomaly detection, enabling dynamic switching of prediction modes or triggering online model fine-tuning. Secondly, explore model lightweighting and deployment optimization by researching techniques such as model pruning and distillation to compress model size and meet the deployment requirements of edge-side devices, while optimizing the parallel computing efficiency of AGA to shorten training cycles. Thirdly, expand multi-station collaboration and multi-source information fusion prediction by researching the extension of the method to collaborative power prediction for multiple energy storage power stations in a region, integrating higher-precision weather forecasts, real-time equipment health status information, etc., to improve prediction accuracy and robustness.

### Abbreviations

**Table:** 

Category	Abbreviation/Symbol	Full Name or Explanation
Models and Algorithms	AGA	Adaptive Genetic Algorithm
	CNN	Convolutional Neural Network
	LSTM	Long Short-Term Memory
	MLP	Multi-Layer Perceptron
	EEMD	Ensemble Empirical Mode Decomposition
	IMF	Intrinsic Mode Function, components obtained from EEMD decomposition
	PFBD	Peak-based Frequency Band Division
Systems and Components	PCS	Power Conversion System (for energy storage)
	SOC	State of Charge
	SOH	State of Health
	EMS	Energy Management System
	MPC	Model Predictive Control
Key Variables and Parameters	$P_{LBatt0}$	Battery pack power loss
	$R_{o}$, $R_{p}$	Ohmic internal resistance, polarization internal resistance of the battery
	$\alpha_{con}$, $\alpha_{SOC}$, $\alpha_{T}$	Series composition of battery pack losses, SOC, operating state correction coefficients
	$\eta_{Batt}$, $\eta_{sub}$, $\eta_{PCS}$	Efficiencies of battery pack, PCS, and energy storage subsystem
	$P_{Closs}$, $P_{Iloss}$	Transformer copper losses, iron losses
	*m, τ*	Embedding dimension, time delay for phase space reconstruction
	*τ*_*w*_	Time window for phase space reconstruction
Evaluation Metrics	MSE	Mean Square Error
	MAE	Mean Absolute Error
	RMSE	Root Mean Square Error
	R²	Coefficient of Determination
